# Transcriptomic Analysis Unveils Correlations between Regulative Apoptotic Caspases and Genes of Cholesterol Homeostasis in Human Brain

**DOI:** 10.1371/journal.pone.0110610

**Published:** 2014-10-16

**Authors:** Raffaella Picco, Andrea Tomasella, Federico Fogolari, Claudio Brancolini

**Affiliations:** Department of Medical and Biological Sciences, Università degli Studi di Udine, Udine, Italy; Karolinska Institutet, Sweden

## Abstract

Regulative circuits controlling expression of genes involved in the same biological processes are frequently interconnected. These circuits operate to coordinate the expression of multiple genes and also to compensate dysfunctions in specific elements of the network. Caspases are cysteine-proteases with key roles in the execution phase of apoptosis. Silencing of caspase-2 expression in cultured glioblastoma cells allows the up-regulation of a limited number of genes, among which some are related to cholesterol homeostasis. Lysosomal Acid Lipase A (LIPA) was up-regulated in two different cell lines in response to caspase-2 down-regulation and cells silenced for caspase-2 exhibit reduced cholesterol staining in the lipid droplets. We expanded this observation by large-scale analysis of mRNA expression. All caspases were analyzed in terms of co-expression in comparison with 166 genes involved in cholesterol homeostasis. In the brain, hierarchical clustering has revealed that the expression of regulative apoptotic caspases (CASP2, CASP8 CASP9, CASP10) and of the inflammatory CASP1 is linked to several genes involved in cholesterol homeostasis. These correlations resulted in altered GBM (Glioblastoma Multiforme), in particular for CASP1. We have also demonstrated that these correlations are tissue specific being reduced (CASP9 and CASP10) or different (CASP2) in the liver. For some caspases (CASP1, CASP6 and CASP7) these correlations could be related to brain aging.

## Introduction

Caspases were initially discovered as critical enzymes in the control of apoptosis. Quite soon it was evident that, they can supervise additional biological processes, such as inflammation and differentiation [1], [2]. This discovery has granted the dichotomy between apoptotic and non-apoptotic caspases. More recently, it has been observed that caspases controlling apoptosis can also play specific roles unrelated to cell death [3]–[5].

Caspases can be divided into initiator and effector caspases depending on the presence of a long prodomain at their amino-terminal region. Initiator caspases act at the apex of a proteolytic cascade, whereas effector caspases act downstream and are involved in the cleavage of specific cellular proteins [1]. Caspase-2, caspase-8, caspase-9, and caspase-10 are the long prodomain caspases involved in the apoptotic process. Caspase-8 and caspase-10 have well-established roles in the engagement of the extrinsic pathway, whereas caspase-9 is the critical enzyme for the intrinsic/mitochondrial pathway. Caspase-8 can also play roles unrelated to apoptosis, such as in NF-kB activation or in limiting necroptosis and caspase-10 has been recently shown to control autophagy [6].

Caspase-2 is still a mysterious caspase. It shows a peculiar nuclear localization that is regulated by two different NLSs [7]. A CARD domain, at the amino-terminal region is responsible for the homotypic interaction with adaptor molecules. Although a molecular platform controlling its activation has been described [8], [9], the contribution of caspase-2 to apoptosis is still debated. Different, sometimes controversial results have been published and multiple functions have been attributed to caspase-2.

Mice deficient for caspase-2 and for its adaptor protein RAIDD have proved absent or only very limited defects in apoptosis [8], [9]. Further studies with cells from caspase-2 −/− mice have indicated that caspase-2 could be considered a tumor suppressor, since its absence can favor oncogene-mediated transformation [10], [11].

Difficulties in defining a gene function in specific biological context could arise from the presence of regulative circuits that compensate the experimental alteration. There are several examples of genes down-regulated by siRNA approaches, or through homologous recombination in mice, which have generated only minimal phenotypes [12]–[14].

The still obscure impact of caspase-2 on cell functions could be masked by compensatory mechanisms engaged following its inactivation. In this manuscript we have investigated the gene expression profile of human cells silenced for caspase-2 expression. Our goal was to unveil whether perturbation of caspase-2 levels could influence the expression of genes involved in specific cellular functions, either as part of common regulative circuits or of compensatory mechanisms.

## Materials and Methods

### Cell culture, siRNA, reagents and antibodies

U87MG and IMR90-E1A cells were grown in DMEM supplemented with 10% FBS, penicillin (100 U/mL), glutamine (2 mmol/L), and streptomycin (100 µg/mL) at 37°C in 5% CO_2_ atmosphere. RNA oligos for interference (RNAi) were purchased from Dharmacon: CASP2 RNAi1, AAACAGCUGUUGUUGAGCGAA; Control1 (CASP2 mutated) RNAi, AAACAAUUGUUGUUGAGCGAA; or Qiagen: CASP2 RNAi2 CAUCUUCUGGAGAAGGACATT and a non-targeting siRNA UUCUCCGAACGUGUCACGU, Control2. Cells were transfected 24 hours after plating by adding the Opti-MEM medium containing Lipofectamine 2000 (Invitrogen) plus RNAi oligos. BSA and Filipin (Sigma), anti-LBPA [15], anti-transferrin receptor (Tnf-R) (OKT9), anti-GM-130 (BD biosciences), anti-caspase-2 [16]. Secondary anti-mouse and anti-rabbit antibodies were Alexa Fluor 488 and Alexa Fluor 546 conjugated (Invitrogen).

### Western blotting

Proteins obtained after an SDS denaturating lysis and sonication were transferred to a 0.2-µm-pore-sized nitrocellulose membrane and incubated with the specific primary antibodies. After several washes, blots were incubated with peroxidase-conjugated goat anti-rabbit or (Euroclone Milano I) for 1 h at room temperature. Finally, blots were developed with Super Signal West Dura, as recommended by the vendor (Pierce).

### RNA expression array and data analysis

Total RNA was isolated using RNeasy Mini kit (Qiagen). RNA sample was labeled according to the standard one cycle amplification and labeling protocol (Affymetrix). Labeled cRNA was hybridized on Affymetrix GeneChip Human Gene 1.0 ST Array. Robust Multi-Array Average (RMA) normalization was applied [17]. Data analysis was performed using the t-test as implemented in the R statistical package. A minimum standard deviation was assumed corresponding to the median percentile of all standard deviations in order to avoid fortuitously large t statistics. Differentially expressed genes were selected based on>1.5 fold change and P<0.05. The analysis of Gene Ontology terms was performed using the DAVID server [18], [19]. Microarray data have been deposited in NCBI Gene Expression Omnibus (GEO) and the GEO accession number is GSE61388.

### RNA extraction and quantitative qRT-PCR (quantitative reverse transcription polymerase chain reaction)

Cells were lysed using Tri-Reagent (Molecular Research Center). A total of 1µg of total RNA was retrotranscribed by using 100 U of Moloney murine leukemia virus reverse transcriptase (Invitrogen). Quantitative reverse transcription-PCR (qRT-PCR) analyses were performed using Bio-Rad CFX96 and SYBR Green technology. The data were analyzed by use of a comparative threshold cycle using HPRT (hypoxanthine phosphoribosyltransferase) and β-actin as normalizer genes. All reactions were done in triplicate.

### Immunofluorescence microscopy

Cells were fixed with 3% paraformaldehyde and permeabilized with 0.5% Triton X-100 and blocked in PBS 3% BSA for 1 h RT. After washes coverslips were incubated with Filipin (100µg/mL) and relative primary antibodies for 2 hrs. After several washes coverslips were incubated with secondary antibodies. Cells were imaged with a Leica confocal scanner SP equipped with a 488 λ Ar laser and a 543 to 633 λ HeNe laser. Cell images for deconvolution were taken using the Leica AF6000 LX microscope.

### Data preparation and analysis

29 human microarray datasets were included in this study, totaling 726 arrays. Brain (11 datasets 293 microarrays) GBM (13 datasets 327 microarray) Liver (5 datasets 106 microarrays) were used and in all cases. All datasets were downloaded manually from GEO [20] and ArrayExpress databases [21]. We analyzed only expression data obtained using the most comprehensive human expression platform HG U133 Plus 2.0. For GBM (GSE11100; GSE13041; GSE15824; GSE19728; GSE23806; GSE23935; GSE29796; GSE30563; GSE32374; GSE4290; GSE7696; GSE9171) for normal human brain (GSE5281; GSE7307; GSE17612; GSE21935; GSE15824; E-MEXP-2351; GSE21354; E-MEXP-2280; GSE15209; GSE7692; GSE4290) for human liver (E-GEOD-40873; E-MTAB-950; E-GEOD-23343; E-AFMX-11; E-MEXP-2128). We processed all the CEL files together by using standard tools available within the affy package in R [22].

We use a UniGene ID centered CDF (Chip Description file) in order to have only one intensity value per gene. CDFs were downloaded from the Molecular and Behavioral Neuroscience Institute Microarray Lab (URL:http://brainarray.mbni.med.umich.edu/Brainarray/Database/CustomCDF/genomic_curated_CDF.asp) [23]. All annotation information were downloaded from the same website. The normalization step was done with the standard MAS5.0 algorithm, described in the Statistical Algorithms Description Document available from Affymetrix (URL: http://www.affymetrix.com/support/technical/whitepapers/sadd_whitepaper.pdf).

We converted all microarray data to log values. We extracted the data regarding the genes of interest in an automatic way. Correlations among gene expression levels were calculated using the library psych in R choosing the Pearson correlation method. p-values were adjusted for multiple testing using Benjamini and Hochberg's method [24]. Genes were clustered using hierarchical clustering using the complete linkage method according to similarity in correlation patterns, as measured by euclidean distance [25]. Heat maps were generated with R with positive correlation scores (values) colored by blue while negative ones colored by dark green. GO (Gene Ontology) annotations and knowledge from the literature was used to create a list of genes involved with the cholesterol metabolism. Gene expression levels were correlated with age using the Spearman's correlation making minimal assumptions about the relationship between the two diverse variables.

## Results and Discussion

### Gene expression profile studies in cells with down-regulated caspase-2 expression

To identify genes and pathways under the influence of caspase-2 we silenced its expression in the glioblastoma cell line U87MG. We selected glioblastoma cells since important apoptotic functions have been attributed to caspase-2 in the brain and because CASP2 deficits elicit compensatory mechanisms in this tissue [26], [27].

**Figure 1:**
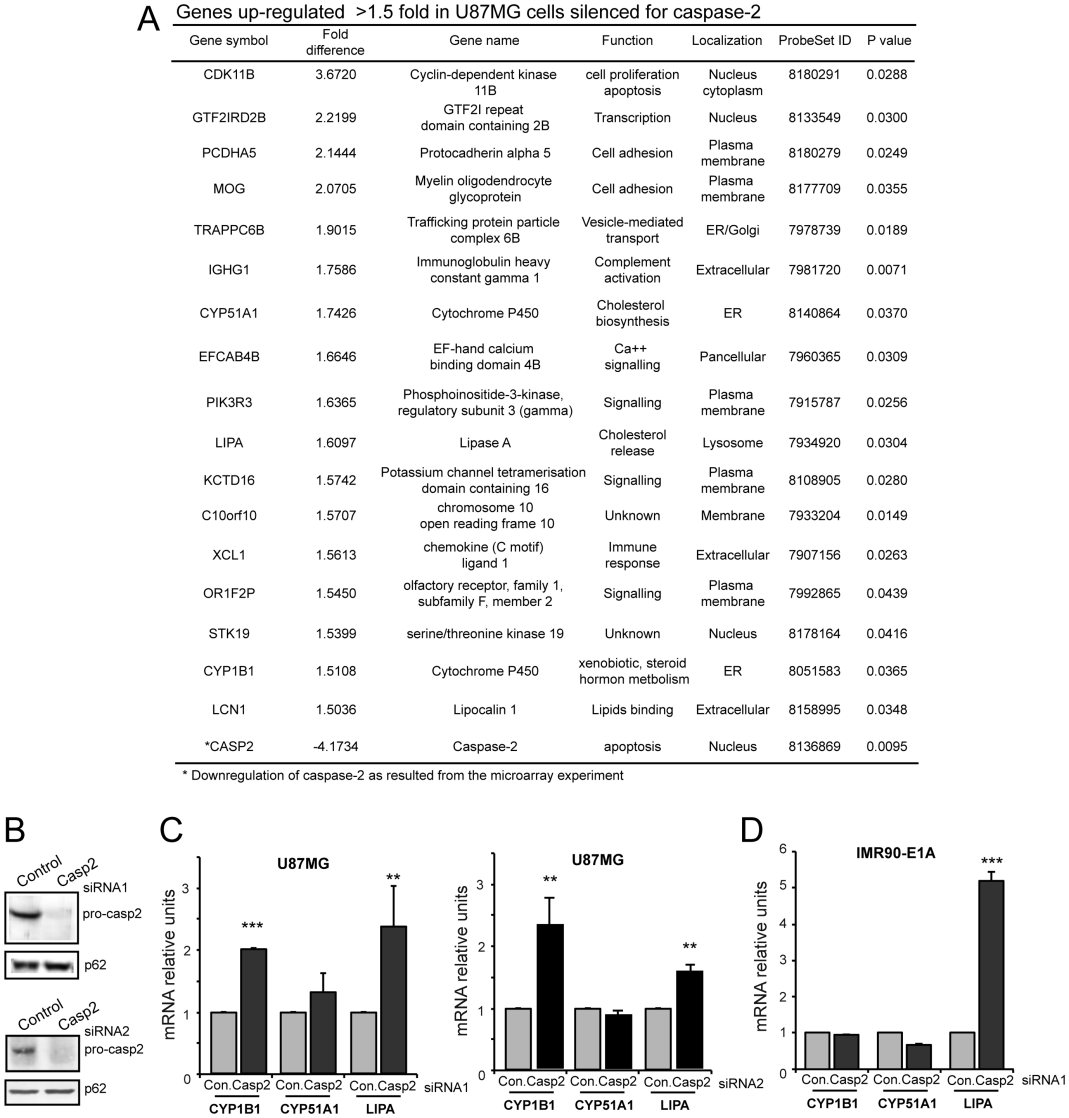
Transcriptomic variations in cells silenced for caspase-2. A. List of the top up-regulated genes (cut-off 1.5) in U87MG cells silenced for caspase-2 expression. B. Cellular lysates from U87MG cells transfected with the indicated siRNAs against caspase-2 or the relative control siRNAs were generated and after immunoblot were probed with an anti-caspase-2 antibody. P62, nucleoporin was used as loading control. C. mRNA expression levels of CYP1B1, CYP51A1 and LIPA were measured using qRT-PCR in U87MG cells transfected with the indicated siRNAs against caspase-2 or the relative control siRNAs. Data are presented as mean ± SD; *n* = 3. D. mRNA expression levels of CYP1B1, CYP51A1 and LIPA were measured using qRT-PCR in IMR90-E1A cells transfected with siRNA1 against caspase-2 or the control siRNA. Data are presented as mean ± SD; *n* = 3.

Caspase-2 deficient cells did not display overt alterations in terms of cell proliferation, cell cycle and apoptosis (data not shown). Next transcriptional expression profiles of cells transfected with caspase-2 siRNA and control siRNA were compared. We selected a 1.5 fold cut-off and globally 24 genes were significantly down-regulated (Table S1), whereas 17 genes were significantly up-regulated in caspase-2 silenced cells (Fig. 1A). This number is particularly small, since in parallel experiments after silencing of other genes, such as USP34 or PGAM5 fluctuations in the expression of more than 200 and 800 genes, respectively were observed (data not shown). Microarray and immunoblot analysis proved the effective down-regulation of CASP2 mRNA (Fig. 1A) and protein (Fig. 1B).

Next we focused our attention on the 17 genes up-regulated after caspase-2 down-regulation, which could be entangled in compensatory responses. Analysis of associated GO terms indicated that these genes are involved in different biological functions, including cell cycle control, inflammation and membrane trafficking. Particularly, 3 of them: CYP51A1, CYP1B1 and LIPA are linked to cholesterol metabolism. Since caspase-2 expression can be influenced by SREBPs and a previous study proposed a role of caspase-2 in the control of cholesterol and triacylgycerol levels [28], [29], we investigated in more detail the relationships between caspase-2 and cholesterol genes.

CYP51A1 and CYP1B1 are cytochrome P450 family members involved respectively in cholesterol/sterol biosynthetic processes and in the metabolism of a wide range of structurally diverse substrates, including cholesterol [30], [31]. LIPA encodes for the key enzyme responsible for acidic hydrolysis of cholesteryl esters and triglycerides delivered from lipoproteins to lysosomes [32].

Furthermore also PIK3R3 (phosphatidylinositol-3 kinase regulatory subunit p55γ), LCN1 (tears lipocalin), other two caspase-2 influenced genes are in some relations with cholesterol and lipid metabolism. The first is a target of SREBPs [33] and the second can bind an assortment of lipids including cholesterol [34]. For all these reasons we decided to study the relationships between caspase-2 and CYP51A1, CYP1B1 and LIPA.

qRT-PCR analysis was performed to validate the microarray experiments. The expression of CYP1B1 and LIPA was augmented in U87MG cells silenced for caspase-2, whereas for CYP51A1 the increase was minimal and not statistically significant (Fig. 1C). To confirm these results we used a second siRNA against caspase-2 (siRNA2) and a second control oligos, from a different provider (Fig. 1B). The results were similar. CYP1B1 and LIPA expression was augment in U87MG cells silenced for caspase-2. We also investigated whether this up-regulation could be observed in other cell lines. Figure 1D shows that only LIPA induction can be observed in human fibroblasts expressing the E1A oncogene after caspase-2 silencing. In this cell line CYP51A1 and CYP1B1 mRNA levels resulted unchanged. However LIPA up-regulation was much more pronounced compared to U87MG cells. These findings indicate that correlations between caspase-2 and cholesterol genes expression could vary in different cell types.

LIPA generates unesterified cholesterol, which can be used as substrate for steroidogenesis or be re-esterified for storage in lipid droplets by acyl-CoA:cholesterol acyl transferase [32]. The increase of LIPA levels in cells with down-regulated caspase-2 might compensate a deficit in Srebp2-driven lipid synthesis/accumulation in human cells, as previously suggested [29].

To understand whether mRNA levels of LIPA, CYP51A1 and CYP1B1 are influenced by other caspases, we silenced caspase-8 expression in U87MG cells. We selected caspase-8 because, like caspase-2, it is a regulative apoptotic caspase, and because it is expressed in U87MG cells (Figure S1A). The designed siRNA silenced caspase-8 expression (Figure S1B). When Caspase-8 expression was down-regulated only CYP1B1 mRNA levels were clearly augmented. LIPA and CYP51A1 expression was not significantly changed.

### Analysis of intracellular cholesterol distribution in caspase-2 silenced cells

**Figure 2:**
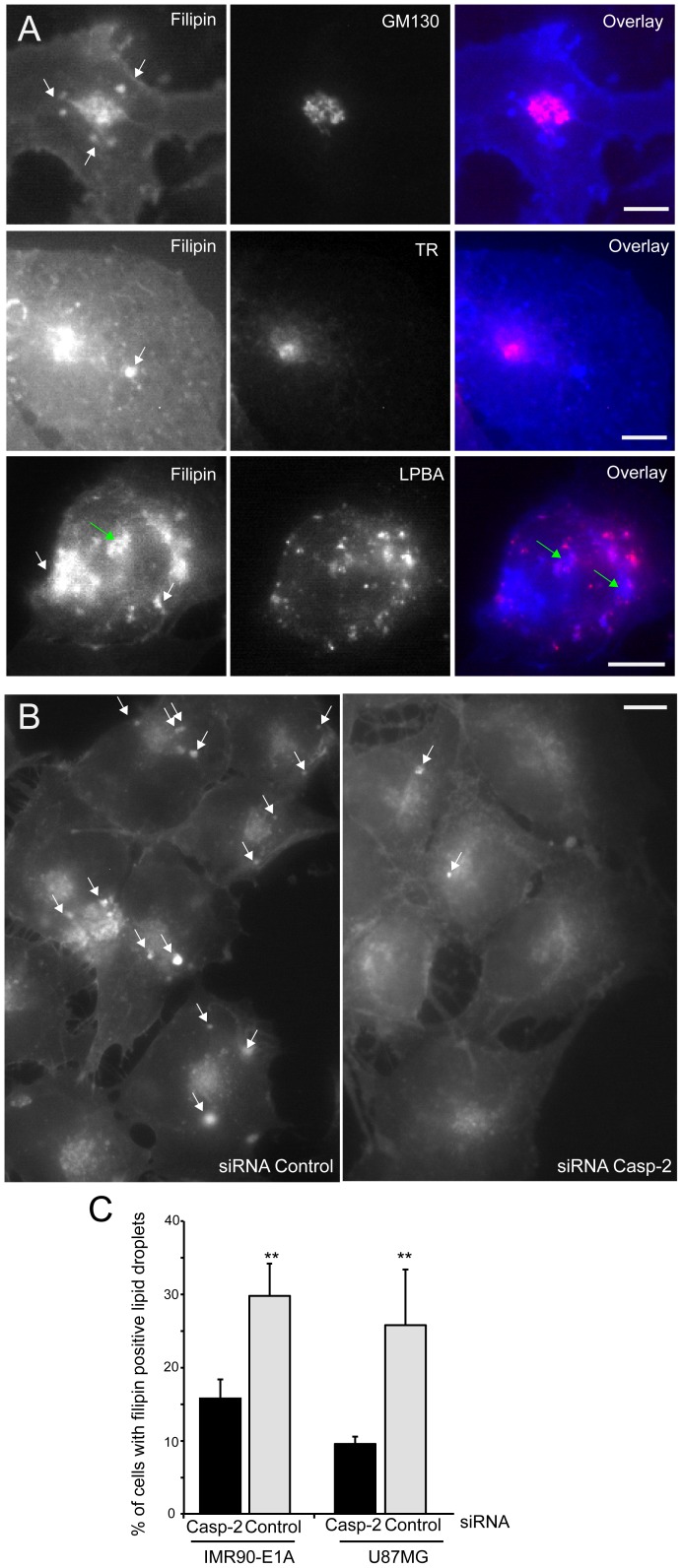
Cholesterol distribution in cells silenced for caspase-2 expression. A. U87MG cells were fixed and processed for immunofluorescence. Epifluorescence microscopy followed by deconvolution analysis was used to visualize different subcellular compartments and cholesterol distribution. With arrows indicate LDs and green arrows LDs encircled by lysosomes. Scale bar 40 µM. B. U87MG cells were transfected with siRNA against caspase-2 or control siRNA as indicated. 48 h later cells fixed and stained with filipin to visualize the intracellular distribution of cholesterol. Arrows point to LDs. Scale bar 30 µM. C. Quantitative analysis of LDs presence in U87MG and IMR90-E1A cells transfected with siRNA against caspase-2 or control siRNA. Data are presented as mean ± SD; *n* = 3.

Having evidences of the existence of a connection linking caspase-2 to some genes of the cholesterol pathway, we compared the subcellular distribution of cholesterol after labeling of IMR90-E1A and U87MG cells with filipin. Fluorescence staining was detectable in the plasma membrane (PM) and in intracellular membrane structures (Fig 2A). As expected co-localization studies using GM130, transferrin receptor and LBPA, as markers respectively of, Golgi apparatus, late endosomal compartment and endosomal/recycling compartment evidenced that cholesterol can be detected in all these organelles. In addition, intense filipin fluorescence staining was present in regular spherical structures that do not co-localize with the used markers and that can be identified as LDs (lipid droplets).

Next we compared filipin staining between caspase-2 silenced and control cells. Remarkably, the number of LDs detectable in a cell, as well as the percentage of cells holding them was reduced after caspase-2 silencing (Fig. 2B). Quantitative analysis proved that in both cell lines, the percentage of cells presenting LDs was reduced after caspase-2 silencing (Fig. 2C). This observation further indicates that down-regulation of caspase-2 could influence in cholesterol homeostasis.

### Correlation studies of LIPA and caspase-2 expression in glioblastoma

**Figure 3:**
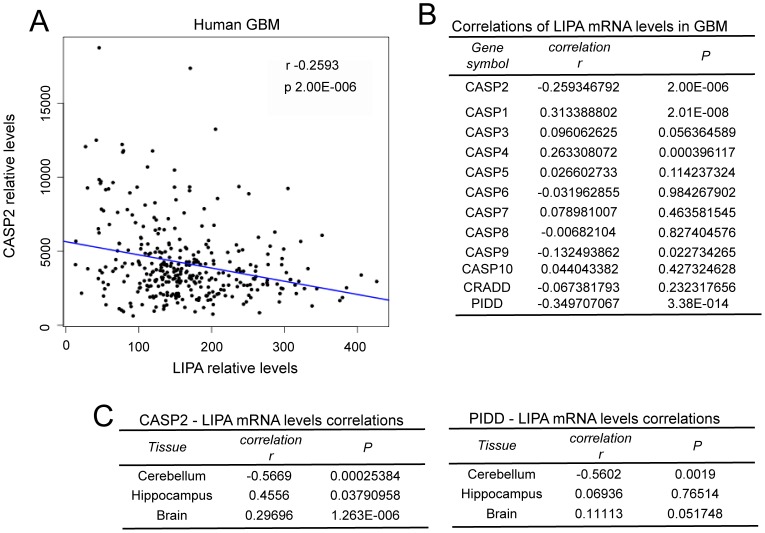
Analysis of LIPA and caspases expression in GBM and different areas of the CNS. A. Plot of CASP2 versus LIPA expression levels in GBM. Linear regression is reported. B. Correlations in expression levels between LIPA and the indicated genes in GBM. C. Correlations in expression levels between LIPA and the indicated genes in different CNS areas.

Taking into account the complex regulative networks influencing cholesterol homeostasis in vivo, the use of the cell culture models to investigate correlations between caspase-2 and cholesterol genes expression is limiting. Hence, to further expand our study we decided to interrogate public available gene expression datasets of glioblastomas. In principle, if caspase-2 controls a circuit that influence LIPA expression in cultured glioblastoma cells the expression of these two genes should be inversely correlated in tumors. Gene expression profiles from 13 datasets including 327 microarrays of GBM were interrogated. Figure 3A illustrates that in glioblastoma, mRNA levels for CASP2 and LIPA evidence a weak, but significant inverse Pearson correlation (r -0.2593; p-value 2.00E-00.6).

Since also caspase-8 down-regulation can influence the expression of certain cholesterol genes (Figure S1), we extended this study to all caspases. as well as, to elements of the molecular platform involved in caspase-2 activation (CRADD/RAIDD and PIDD) [8], [9]. With the exclusion of CASP1 mRNA, which level weakly but positively correlates with LIPA, all the other caspases do not evidence significant correlations with LIPA expression (Fig. 3B). Concerning PIDD, its expression negatively correlates with LIPA mRNA levels (r -0.3497; p-value 3.38E-014) in GBM.

Expression correlations between CASP2/LIPA and PIDD/LIPA were also evaluated in normal tissues. A good and significant inverse correlation was scored for both genes in cerebellum. On the contrary, in the hippocampus and with lowest score in the brain, a significant positive correlation only for CASP2/LIPA was observed (Fig. 3C). Interestingly, differences in terms of expression of cholesterol genes between different brain areas are known [35].

### Expression correlations among caspases and cholesterol genes in normal brain and in glioblastoma

Although in cultured cell lines we noted an inverse correlation in terms of expression between CASP2 and LIPA, in vivo the situation is heterogeneous, as suggested by the analysis of different CNS (Central Nervous System) areas. Moreover, since silencing of caspase-2 affects also other cholesterol genes, it is evident that these correlations cannot be simply analyzed without taking into account other genes involved in cholesterol homeostasis. For example down-regulation of LIPA provoked the up-regulation of several genes implicated in cholesterol biosynthesis, as compensatory mechanism [36]. Therefore, we decided to investigate with a more comprehensive approach the correlations between expression of caspase-2 and the expression of genes involved in cholesterol homeostasis. We extended this study to all caspases and we also included the normal tissues. Gene expression profile data from 11 datasets including 293 microarrays of normal brain were recovered from public available databases.

**Figure 4:**
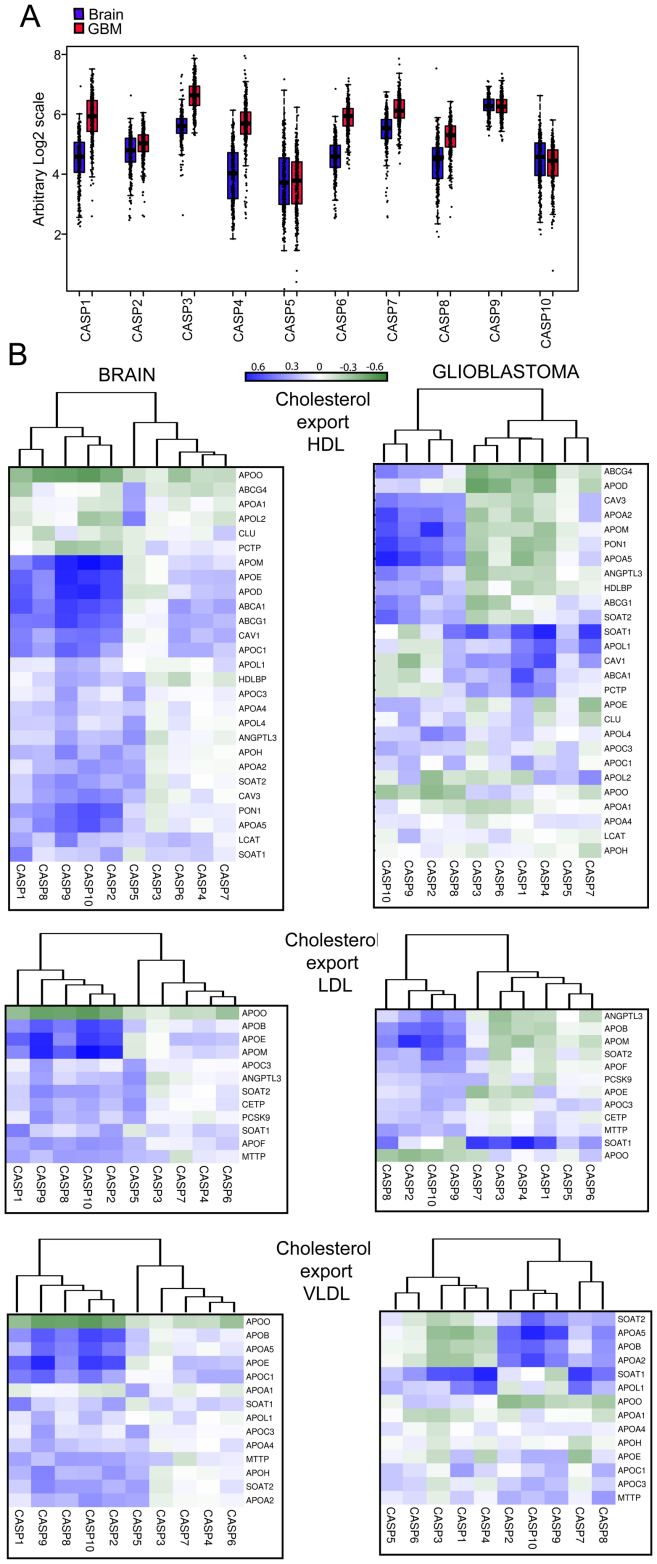
Co-expression analysis of caspases and cholesterol genes in human brain and GBM. A. Expression levels of the different caspases in GBM and in the normal brains. Box plots depicted in red mark tumors whereas blue was used for normal brain. mRNA levels of CASP1, CASP3, CASP4, CASP6, CASP7 and CASP8 were significantly augmented in tumor samples. p-value <2.2^e-16^. B. Correlations of expression levels between caspases and cholesterol genes in different brain and GBM samples in *Cholesterol export, lipoproteins* category. Data obtained were used to calculate the correlation values with the Pearson method. In the heat map positive values are displayed in blue and negative in dark green. The dendrograms displayed on the top are based on hierarchical clustering using the complete linkage method.

We first evaluated changes in the mRNA levels for the different caspases between normal brain and GBM. Surprisingly the mRNA levels of several caspases, both inflammatory (CASP1 and CASP4) and apoptotic (CASP3, CASP6, CASP7 and CASP8) were significantly augmented in tumor samples (Fig. 4A).

Next, genes involved in cholesterol homeostasis, including steroidogenesis were extracted from Gene Ontology (GO) and integrated from literature data. In total we selected 256 genes, of which 166 were grouped into 5 categories: *biosynthesis*, *adsorption/import*, *export*, *steroid and bile acid synthesis* and *transcriptional regulators*. The category *export* was subdivided into HDL (High Density Lipoproteins), LDL (Low Density Lipoproteins) and VLDL (Very Low Density Lipoproteins). At this point we exploited microarray datasets to evaluate correlations in the expression levels between caspases and cholesterol genes. Pearson correlation between the expression level of each caspase and each cholesterol gene was computed for human brain samples. Based on the similarity in correlation patterns caspases and cholesterol genes were hierarchically clustered using the complete linkage method.

#### Cholesterol export, lipoproteins

The CNS produces its own lipoprotein transport system that is distinct from the plasma [37]. CASP1, 2, 8, 9, and 10 share correlations in terms of changes in the mRNA levels with several genes involved in HDL biogenesis. In particular expression of CASP2, 9 and 10 show the highest correlations with APOD, APOM, APOE, APOA5, PON1, CAV1, CAV3, ABCA1 and ABCG1 (Fig. 4B and Table S2).

APOD and APOM are apolipoprotein belonging to the lipocalin protein superfamily [38]. APOM expression is under the influence of hormones and cytokines [39]. In *apoM*-deficient mice plasma HDL are reduced by approximately 17–21% [40]. Moreover, APOM is an important carrier of shingosine-1-phosphate (S1P), a signaling molecule controlling several processes including inflammation [41]. APOD not only contributes to HDL formation but can also act as antioxidant [42]. This function could explain the up-regulation of APOD expression with aging in human prefrontal cortex, as part of a protective circuit [38], [43].

APOE is the major apolipoprotein for CNS HDL and is mainly synthetized by astrocytes [37], [44]. It is possible that ApoE-containing lipoproteins are involved in delivering cholesterol to neurons for growth, repair and synaptogenesis [37], [45]. Fluctuations in APOE mRNA could be related to repair. In fact, in astrocytes ApoE synthesis increases dramatically after nerve injury [46], [47]. APOE and in particular the ε4 allele is the major known genetic risk factor for late-onset Alzheimer's disease [48].

PON1 belongs to paraoxonase genes family and is secreted into the extracellular environment where it binds HDL. This antioxidant enzyme confers to HDL some of the anti-atherogenic properties, such as HDL-mediated cholesterol efflux from macrophages, and the inhibition of LDL oxidation [49].

Although in the brain their activities are less characterized, ATP-binding cassette (ABC) transporters ABCA1 and ABCG1, but also CAV1 and CAV3 mediate cholesterol efflux and play important roles in the transfer of phospholipids and cholesterol to apolipoproteins such as ApoE and ApoM [37], [50].

In GBM expression correlations among constituents of HDL and pro-apoptotic regulative CASP2, 8, 9, and 10 are still present but reduced. In particular CASP2, 9 and 10 do not show significant correlations with APOD, ABAC1 and CAV1 and also in the case of ABCG1 the correlation is diminished. Instead, another transporter, ABCG4 exhibits correlations.

In GBM, CASP1 reveals profound different correlations with HDL genes and clusters together with CASP4, another inflammatory caspase, and the effector caspases. HDL genes showing strongest correlations with CASP1 are APOL1, SOAT1, CAV1, ABCA1 and PCTP. Interestingly, cholesterol efflux associates more strongly with the expression of ABCG1 than of ABCA1 [51].

#### Cholesterol biosynthesis

**Figure 5:**
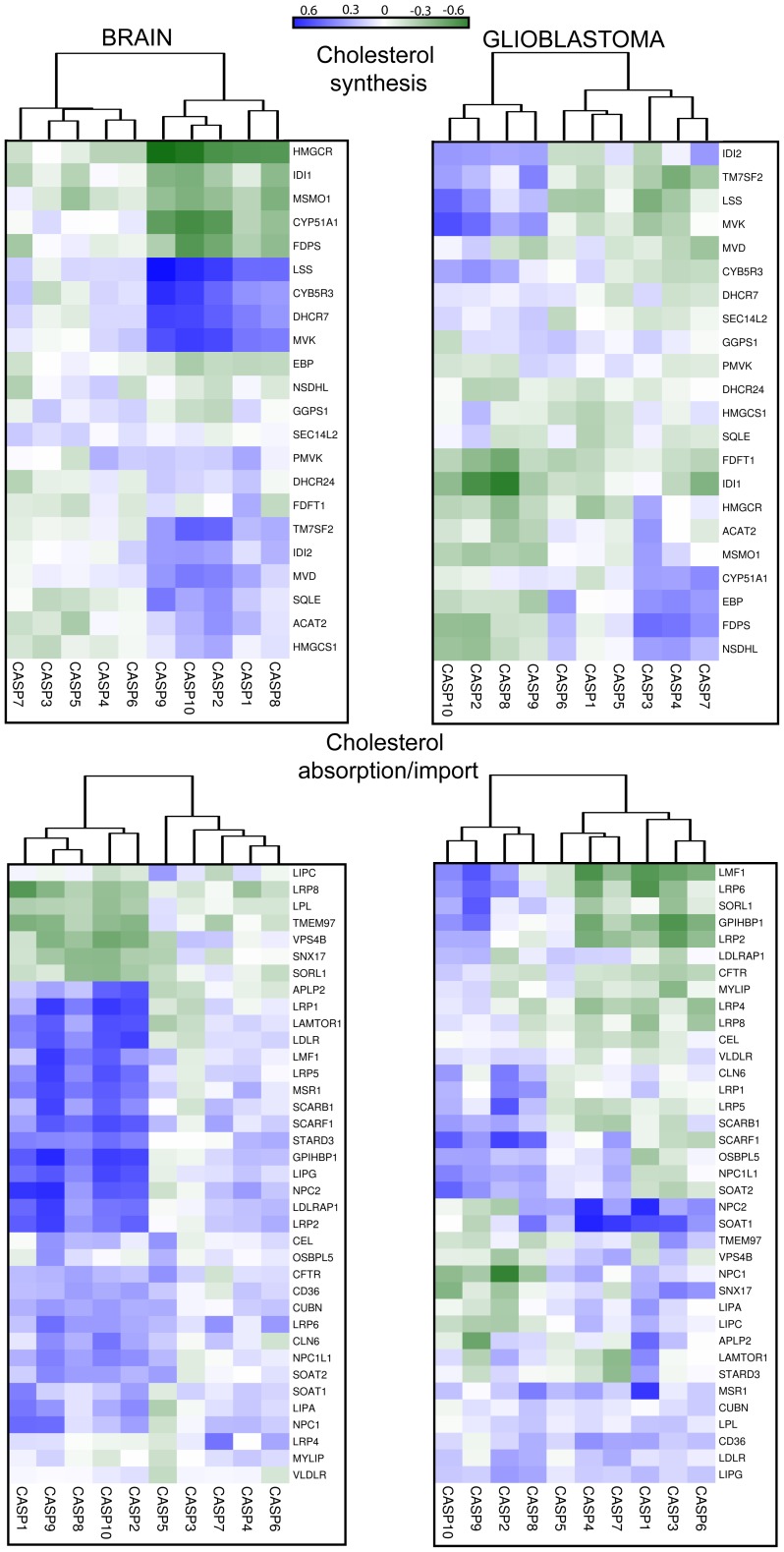
Co-expression analysis of caspases and cholesterol genes in human brain and GBM. Correlations in terms of expression levels between caspases and cholesterol genes in different brain and GBM samples, for the indicated categories. Data obtained were used to calculate the correlation values with the Pearson method. In the heat map positive values are displayed in blue and negative in dark green. The dendrograms displayed on the top are based on hierarchical clustering using the complete linkage method.

Almost all the cholesterol found in the CNS is produced from local biosynthesis [52]. Also in the case of the category “cholesterol biosynthesis”, correlation analysis revealed that the regulative apoptotic caspases, with the addition of CASP1 cluster together. In particular strong positive correlations (Fig. 5 and Table S3) with mRNA levels of a group of genes involved in cholesterol biosynthesis (LSS, CYB5R3, DHCR7, MVK) emerged. By contrast, HMGCR the gene encoding for the enzyme converting the 3-hydroxyl-3-methyl-glutarylCoA (HMG-CoA) into mevalonate scores a good but negative correlation. Since it represents the limiting step in cholesterol biosynthesis, the implications of the correlations between certain enzymes of cholesterol biosynthesis and regulative apoptotic caspases are unclear at the moment. Significant correlations were not observed for all the other caspases. Expression of CYP51A1 in the brain achieves a strong inverse correlation compared to CASP2, CASP9 and CASP10. CYP51A1 encodes for lanosterol 14α-demethylase, which in addition to being a key enzyme of the cholesterol biosynthetic pathway [31] is also involved in the steroidogenesis [53].

In GBM correlations among regulative apoptotic caspases and genes involved in cholesterol biosynthesis are less evident. Again CASP1 clustered with effector caspases in the absence of significant correlation scores. Interestingly, CASP3 shows good correlations with some genes involved in cholesterol biosynthesis including HMGCR.

#### Cholesterol absorption/import

Lipoproteins produced by astrocytes can be internalized, after binding to LDL receptor superfamily by neurons and glia cells [37]. Also in the category of absorption/import, analysis of the correlations at mRNA levels resulted in two clusters where the regulative apoptotic caspases plus CASP1 segregate from all the others. CASP2 CASP9 and CASP10 show the strongest correlations with APLP2, LRP1, LDLR, LRP2, LRP5, MSR1, LIPG, SCARF1, GPIHBP1, NPC2, LAMTOR1 and LDLRAP1 (Fig. 5 and Table S4).

LDLR, LRP1, LRP2, LRP5 are all members of the low-density lipoprotein receptors family [54]. These receptors can bind a large number of extracellular ligands but a common ligand for all is the ApoE protein, which mediates internalization and catabolism of lipoprotein particles [55]. The LDLR is highest expressed in glial cells than in neurons, on the opposite LRP1 is highest expressed in neurons than in glia [56], [57]. These observations suggest that correlations with regulative apoptotic caspases are not limited to a specific cell type. APLP2 an APP homologous can influence LRP1 expression and the toxicity mediated by beta-amyloid oligomers [58]. LDLRAP1 encodes for an adaptor cytosolic protein that interacts with and is involved in the endocytosis of LDLR. MSR1, SCARF1 and SCARB1 are involved in the uptake of lipoproteins. Endothelial lipase (LIPG) regulates the circulating level of HDL [59]. It is expressed in different areas of the CNS including CA3 pyramidal cells of the hippocampus, ependymal cells in the ventral part of the third ventricle [60]. LIPG is also expressed in brain capillary endothelial cells, major constituents of the blood brain barrier [61]. GPIHBP1, (glycosylphosphatidylinositol-anchored high-density lipoprotein binding) can be considered a platform for lipolysis [62] and it is also involved in the transport of lipoprotein lipase (LPL) [63]. LAMTOR1 and NPC2 work downstream to the internalization of lipoproteins. The first as key regulator of endosome dynamics and lysosome biogenesis, the second by removing unesterified cholesterol from late endosomes/lysosomes [64], [65].

It is evident that several genes involved in cholesterol and lipids up-take show a coordinate expression with regulative apoptotic caspases. High positive scores in terms of correlation among pro-apoptotic regulative caspases and genes involved in cholesterol internalization are in agreement with the negative score observed in the case HMGCR [66]. In GBM, as above observed for other categories, these correlations are less robust and CASP1 distinctly changes relationships with cholesterol genes, thus clustering with the effector caspases.

#### Steroid and bile acid synthesis

**Figure 6:**
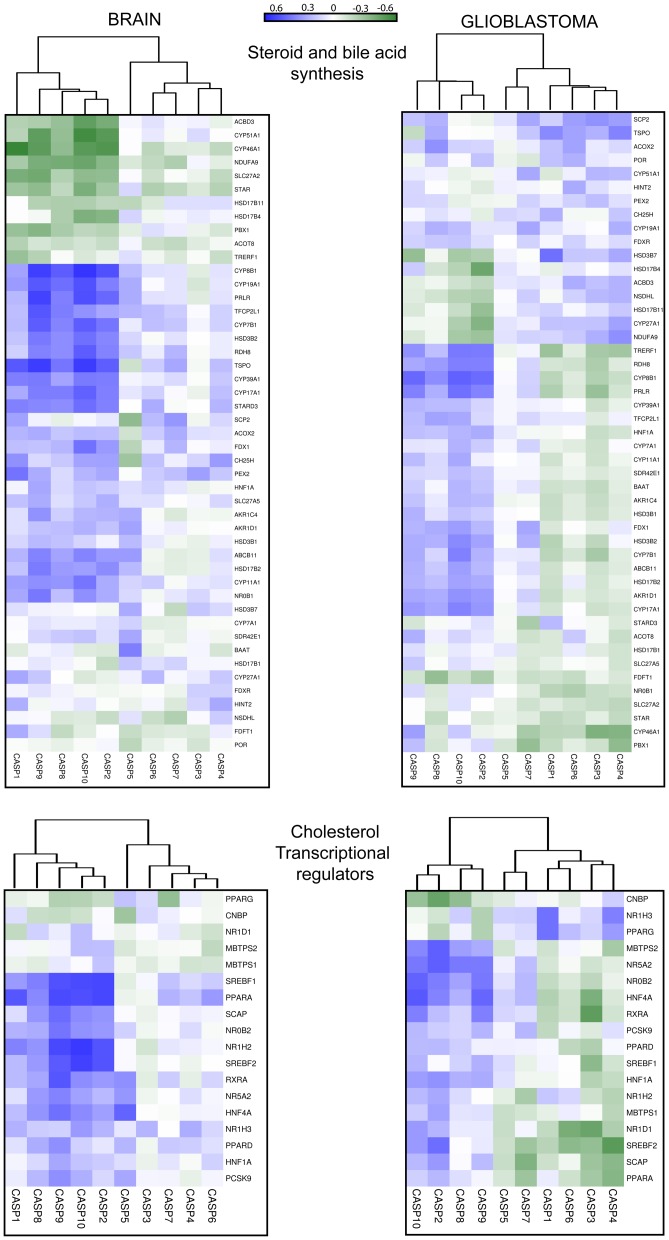
Co-expression analysis of caspases and cholesterol genes in human brain and GBM. Correlations of expression levels between caspases and cholesterol genes in different brain and GBM samples, for the indicated categories. Data obtained were used to calculate the correlation values with the Pearson method. In the heat map positive values are displayed in blue and negative in dark green. The dendrograms displayed on the top are based on hierarchical clustering using the complete linkage method.

This category collects heterogeneous gene families. Nevertheless, the apoptotic regulative caspases cluster again together, whereas CASP1 is here found together with other inflammatory caspases and with the effector caspases. TSPO, CYP7B1, CYP8B1 and CYP19A1 show the highest positive correlation scores (Fig. 6 and Table S5).

Steroid biosynthesis begins with the transfer of free cholesterol from intracellular stores into mitochondria [67]. TSPO encodes for a translocator located on the outer mitochondrial membrane, which binds cholesterol with high affinity and transfers it in the inner mitochondrial membrane. TSPO activity is the rate-limiting step in the synthesis of all steroids [68]. Interestingly, alterations in TSPO expression has been found in various psychiatric disorders, including social phobia, post-traumatic stress disorder, adult separation anxiety and schizophrenia [69].

CYP7B1 catalyzes 7-hydroxylation of C_19_ and C_21_ steroids and in the brain it is involved in the metabolism of neurosteroids and oxysterols [70]. Defects of CYP7B1 in humans have been linked to spastic paraplegia [71], [72].

CYP8B1 is a sterol 12α–hydroxylase. In mice expression of CYP7A1 and of CYP8B1 is integrated [73]. In the liver, circadian signals can influence CYP7A, CYP8B, and CYP51A1 expression [74].

CYP19A1 encodes for the cytochrome P450 aromatase an enzyme responsible for the synthesis of all oestrogens from androgen precursors [75]. Oestradiol synthesis in the brain regulates several functions of the adult CNS, from neural plasticity to injury responses [76]. Increased expression of CYP19A1 during neurodegeneration could interfere with apoptotic pathways and to decrease the extent of brain damage [77].

In glioblastoma the correlations affect other genes and are in general attenuated. TSPO expression is not longer linked to regulative caspases, whereas it shows positive correlations with effectors and inflammatory caspases.

#### Transcriptional regulators

Finally, we investigated the correlations among caspases and the expression of TFs (Tramscription Factors) involved in the control of cholesterol genes expression. Figure 6 documents the results, which photocopy those previously obtained with the other categories. Apoptotic regulative caspases and CASP1 share similar relationships, in terms of expression correlations, with transcriptional regulators of cholesterol genes. SREBF1, SREBF2, PPARA and NR1H2 display the highest score with CASP2, CASP9 and CASP10 (Figure 6 and Table S6). In the case of SREBF2, Pearson correlation coefficients with caspases were similar to those described for cholesterol genes [78].

SREBF1 and SREBF2 (sterol regulatory element binding proteins) are TFs that control cellular lipid homeostasis. SREBF1 encodes for two proteins Srebp1a and Srebp1c produced via alternative transcription start sites. Srebp1c preferentially influences expression of fatty acid biosynthesis genes, whereas Srebp2 is devoted to transcribe genes involved in cholesterol homeostasis, lipoproteins import and lipids trafficking. Serbp1a can support transcription of both SREBF1 and SREBF2 genes [79]. In this respect it is interesting to note that only SREBF2 accomplishes a good correlation with CASP2 in GBM.

PPARA belongs to the family of peroxisome proliferator-activated receptors, which includes (PPARα, PPARβ/δ and PPARγ). These TFs function as obligate heterodimers with retinoid-X receptors (RXRs). PPARα supervises energy homeostasis by stimulating fatty acids and cholesterol breakdown and gluconeogenesis [80]–[82]. PPARβ/δ is mainly engaged in fatty acid oxidation. PPARγ principal activity is to drive storage of lipids, in particular by controlling adipocyte differentiation [83]. In the brain PPARD is the most abundant and quite ubiquitously expressed member, whereas PPARA and PPARG are expressed in more restricted areas and cell types [84], [85]. In addition to their metabolic role, PPARs in the CNS have been implicated in the control of neuronal differentiation, death, inflammation and neurodegeneration [83]. In GBM expression correlation between PPARA and regulative caspases are abrogated. On the other side a good correlation between PPARG and CASP1 appears (Fig. 6).

NR1H2 (liver X receptor-beta) is another master TF orchestrating the expression of genes of the cholesterol homeostasis [86]. Similarly to SREBF1, SREBF2 and PPARA correlations between NR1H2 and CASP2, CASP9 and CASP10 are abrogated in GBM. Interestingly, in GBM new correlations emerged between the second nuclear hormone receptors liver X receptor alpha (LXRalpha/NR1H3) and inflammatory caspases CASP1 and CASP4.

In summary, the correlation among regulative apoptotic caspases and certain cholesterol genes observed in this study could be orchestrated by selected TFs, well-known master regulators of cholesterol metabolism such as SREBF1, SREBF2, PPARA and NR1H2.

### Expression correlations among caspases and cholesterol genes in the liver

**Figure 7:**
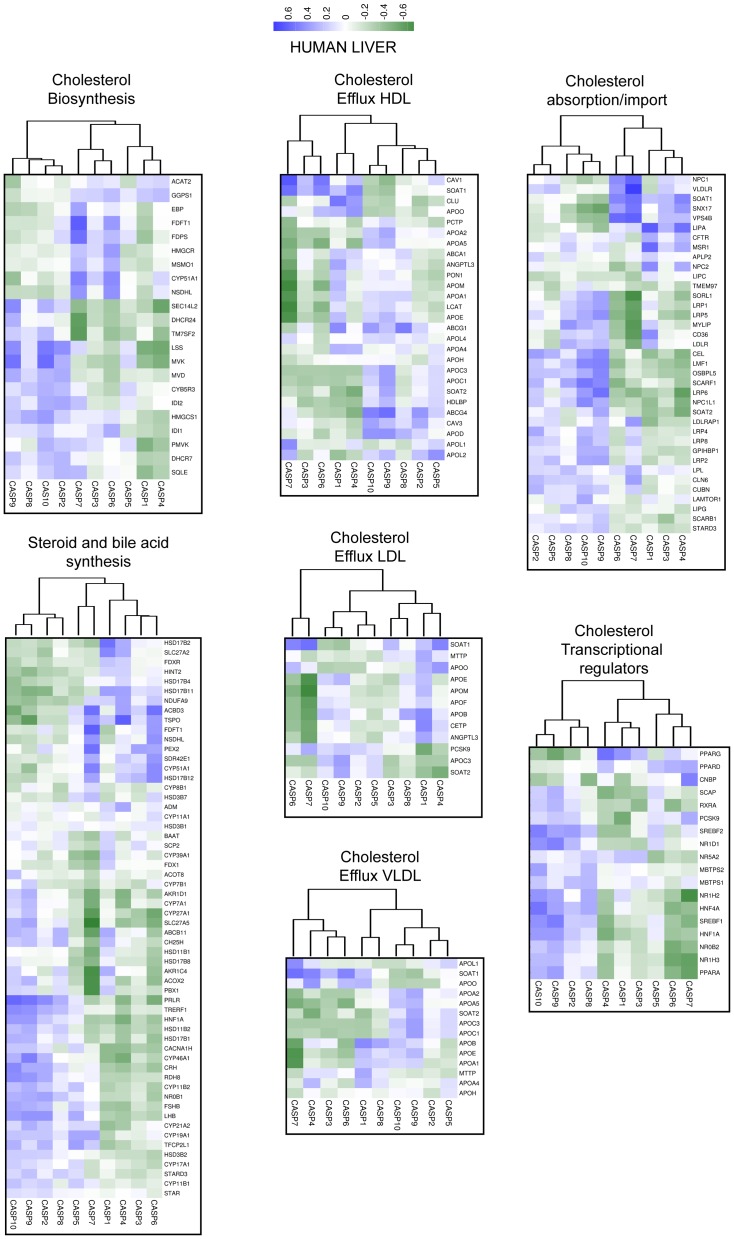
Co-expression analysis of caspases and cholesterol genes in human liver. Correlations of expression levels between caspases and cholesterol genes in different liver samples, for the indicated categories. Data obtained were used to calculate the correlation values with the Pearson method. In the heat map positive values are displayed in blue and negative in dark green. The dendrograms displayed on the top are based on hierarchical clustering using the complete linkage method.

To understand whether our discoveries are limited to CNS or can be observed also in other tissues, we decided to compare variations in the expression levels of caspases and of cholesterol genes in human liver, an essential organ for cholesterol homeostasis. Gene expression profiles from 5 datasets including 106 microarrays of normal human liver were interrogated. Figure 7 shows that correlations among cholesterol genes and expression of regulative apoptotic caspases are less pronounced and only in some categories: *transcriptional regulators*, *cholesterol biosynthesis*, *steroid and bile acid synthesis*, these genes cluster together. In the liver CASP7 reaches the highest scores both for positive and negative correlations. For example CASP7 expression is strongly inversely correlated with those of several apolipoprotein genes, but it positively correlates with NPC1, VLDLR, SOAT1, SNX17 and VPS4B (Table S7); which are genes involved in cholesterol up-take and storage. It is important to note that similarly to caspase-2, caspase-7 expression is under the influence of Srebp1/2 and of statins [87].

CASP9 and CASP10 in terms of correlations with cholesterol genes maintain in the liver a pattern resembling that remarked in the brain and they cluster in almost all categories. By contrast CASP2 exhibits a correlation pattern rather different from the brain and it frequently clusters with CASP5. This observation suggests that the correlations between caspase-2 and cholesterol genes are related to a specific brain endeavor [88], [89]. Remarkably, several reports have proposed specific biological activities for caspase-2 in the CNS [26], [89]–[91].

### Caspases, cholesterol genes and aging

**Figure 8:**
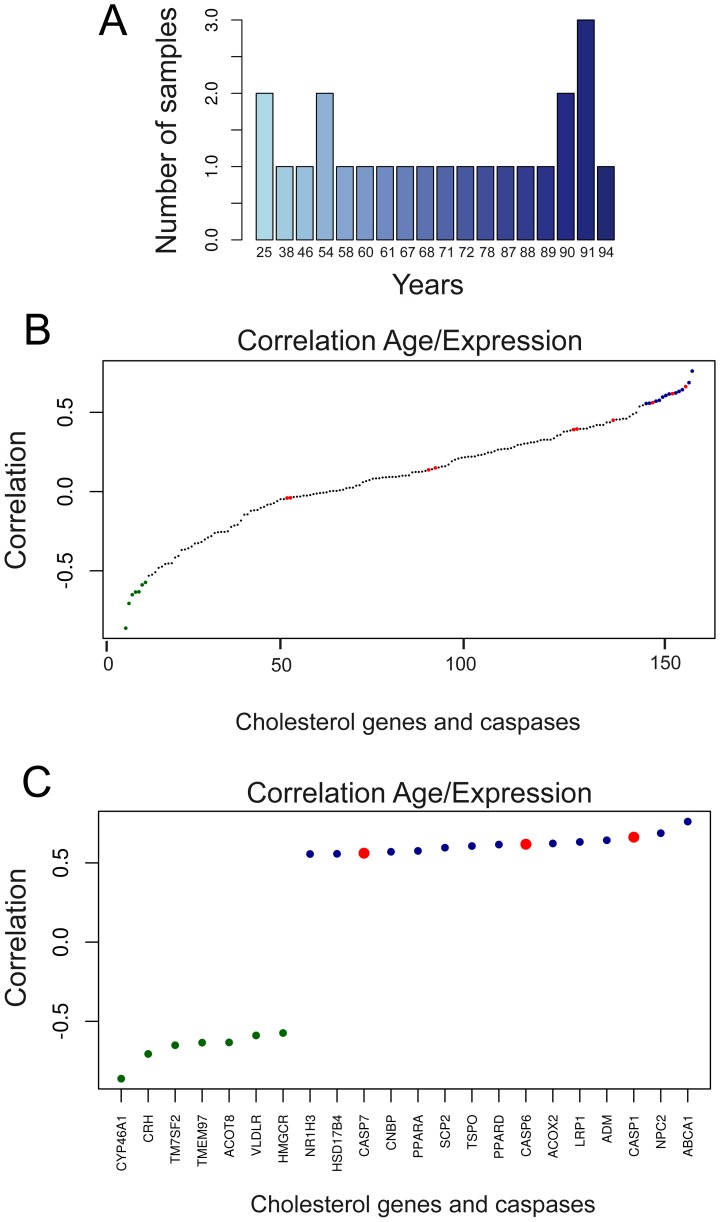
Analysis of cholesterol and caspase genes expression with aging in anterior prefrontal cortex. A. Bar plot displaying the age frequencies of the samples used in the analysis. B. Dot plot of ranked correlations of the 129 genes involved in cholesterol homeostasis/steroidogenesis and caspases respect to age. C. Dot plot of ranked correlations of genes involved in cholesterol homeostasis/steroidogenesis and caspases that scored significant expression correlation with respect to age. We applied the Spearman method (Benjamini and Hockberg correction). P<0.05.

Changes in the expression of cholesterol genes in the brain are linked to several physiological/pathological conditions including, regeneration, plasticity, circadian rhythms, diet, degeneration, social behavior and also the presence of several different cell linages [37], [38], [43], [58], [69], [74], [77], [83], [92]. To understand the significance of the described correlations, we decided to interrogate more precisely the public available datasets used in our studies. We restricted our inquiries to one dataset (GSE 17612) in which, information about the specific brain area (the anterior prefrontal cortex/Brodmann area 10) and subjects age were available. 23 microarray of normal brain met these requirements. Age distribution is shown in figure 8A. Cholesterol and caspase genes were ranked accordingly to the Spearman method, respect to the age of the subjects. Figure 8B illustrates that the expression of a set of cholesterol genes shows a good inverse correlation respect to age (green dots) and on the opposite, the expression of a different set of genes increases with aging (blue dots).

Among genes, which mRNAs levels are down-regulated with aging, we have found CYP46A1 and HMGCR (Fig. 8B and 8C). CYP46A1 encodes for the hydroxylase, which converts cholesterol to 24S-hydroxycholesterol and provides the major route in cholesterol excretion from the brain [93]. In the case of HMGCR our observations are in agreement with previous results pointing to a decline in brain cholesterol synthesis with age [43], [94], [95]. On the opposite previous study reported an up-regulation of cholesterol-24-hydroxylase mRNA in mouse brain with aging [96]. Indeed in their studies the authors analyzed the hippocampus from day 10 up to 21 months. After an up-regulation at 3 months, a trend to decrease in both mRNA and protein levels of CYP46A1 can be appreciated. In humans 24S-hydroxycholesterol levels are highest in the first decade of life and then decline with age [97]. Furthermore, also in human brain, analysis of cholesterol 24-hydroxylase protein evidenced some reduction during aging [98]. Undoubtedly, further studies are necessary to clarify this point. Taking into account that mice lacking 24-hydroxylase exhibit severe learning and memory defects [99], clarification of CYP46A1 expression during aging is of particular interest.

We have included in this analysis also the different caspases. Figure 8C reveals that expression of inflammatory CASP1 and also of the effector caspases CASP6 and CASP7 positively correlates with aging.

Several studies have indicated a contribution of caspase-6 to neurodegeneration including Alzheimer and Huntington diseases [100]–[104]. A more recent report has proposed that caspase-6 activity can predict lower episodic memory ability in aged individuals [105]. CASP1 is a key regulator of inflammation, via the generation of IL-1β and involved in the regulation of age-related cognitive-dysfunctions. CASP1 genetic variations have been associated with cognitive function [106] and several data have linked caspase-1 to brain aging [107], [108]. Deficiency in Nlrp3 inflammasome-mediated caspase-1 activity improved cognitive function and motor performance in aged mice [109]. Furthermore, caspase-1 also influences in a still undetermined manner lipid metabolism [110].

### Conclusions

Our initial aim, of discovering compensatory pathways to solve the caspase-2 mystery has led us to unveil large and shared correlations among different caspases and cholesterol genes. Expression correlations studies have attracted attention to uncover new biological circuits [111], [112] and dedicated methodological tools have been developed [113], [114]. We have applied hierarchical clustering of gene expression correlations to hypothesize new functions for caspases and caspase-2 in particular. An unexpected finding was that the regulative apoptotic caspases (CASP9 and CASP10 in particular) share a correlation pattern with cholesterol genes, similarly to CASP2. The reported strong correlations among these caspases and certain cholesterol genes in a heterogeneous tissue, such as the human brain and in a heterogeneous population, suggest that expression of these genes is influenced by common signaling networks linked to specific biological processes. Hence, expression of certain cholesterol genes and of regulative apoptotic caspases in the brain should be under the control of the same regulative circuits. Since in GBM these correlations are in general less prominent, it is possible that genetic lesions, altering proliferation also impact on cholesterol homeostasis. Although the contribution of cholesterol genes to GBM development is largely unexplored, recently a survival pathway engaged by the LDL receptor, through the EGFRvIII/PI3K/SREBP-1 axis has been discovered [115]. This observation suggests that oncogenic driven changes in gene expression could revise the harmonic co-regulation of caspases and cholesterol genes.

This scenario is well exemplified by CASP1, which evidences the most overt changes in terms of correlations with cholesterol genes between brain and GBM. Taking into account that its expression is augmented in GBM, these changes could reflect the establishment of an inflammatory microenvironment.

When the same analysis were performed using microarray data obtained from cultured glioblastoma cells, correlations were in general weaker and apoptotic regulative caspases clustered separately (data not shown). This observation indicates that complex networks of environmental signals control the expression of these genes. Networks that cannot be easily replaced under *in vitro* culture conditions. A consideration that could explain the low number of genes, which expression is influenced by caspase-2 siRNA in cultured glioblastoma cells.

It is evident that, correlations in terms of expression could have different biological origins/implications: from the presence of different cell lineages in the sample, up to different neuronal activities, different inflammatory/degenerative states, different cognitive functions and different hormonal signaling. We are consciousness that this complexity deserves further experimental work. Here we have discovered that for some caspases, in a specific brain's area, the correlations with certain cholesterol genes could be related to aging. In this case the correlation could be linked to the induction of degenerative processes. In fact, caspase signaling engaged by the ordered activation of caspase-8 and caspase-3/7 controls microglia activation and neurotoxicity with implications in Parkinson's and Alzheimer's diseases [116].

For other caspases, including caspase-2 the reason for this correlation is unclear at the moment. Taking into account that evidences sustaining non-apoptotic roles of caspases in the CNS are accumulating [117] several hypothesis concerning caspase-2 could be formulated. Previous studies [28], here confirmed, linking SREBFs levels to CASP2 expression suggest a direct involvement of this enzyme in cholesterol homeostasis. Although the mechanisms need to be elucidated, our data further encourage investigating towards this direction.

## Supporting Information

Figure S1 A. Expression profiles in U87MG cells of the different caspases. Microarray expression data were obtained from GSE14889 [118]. B. mRNA expression levels of CASP8 were measured using qRT-PCR in U87MG cells transfected with siRNA against Caspase-8 or a control siRNA. Data are presented as mean ± SD; *n* = 3. C. mRNA expression levels of CYP1B1, CYP51A1 and LIPA were measured using qRT-PCR in U87MG cells transfected with siRNA against Caspase-8 or a control siRNA. Data are presented as mean ± SD; *n* = 3.
(JPG)

Table S1 **Genes down-regulated>1.5 fold in U87MG cells silenced for Caspase-2.**
(XLS)

Table S2 **Top score cholesterol genes of the subclass** ***cholesterol export*** **in terms of correlations of expression levels with regulative apoptotic caspases in human brain.** Values are shown for all caspases.
(XLS)

Table S3 **Top score cholesterol genes of the subclass** ***cholesterol biosynthesis*** **in terms of correlations of expression levels with regulative apoptotic caspases in human brain.** Values are shown for all caspases.
(XLS)

Table S4 **Top score cholesterol genes of the subclass** ***cholesterol adsorption/import*** **in terms of correlations of expression levels with regulative apoptotic caspases in human brain.** Values are shown for all caspases.
(XLS)

Table S5 **Top score cholesterol genes of the subclass** ***steroid and bile acid synthesis*** **in terms of correlations of expression levels with regulative apoptotic caspases in human brain.** Values are shown for all caspases.
(XLS)

Table S6 **Top score cholesterol genes of the subclass** ***transcriptional regulators*** **in terms of correlations of expression levels with regulative apoptotic caspases in human brain.** Values are shown for all caspases.
(XLS)

Table S7 **Top score cholesterol genes in terms of correlations of expression levels with caspase-7 in human liver.** Values are shown for all caspases.
(XLS)
