# Supplementary figures and images for: Transcriptomic Analysis Unveils Correlations between Regulative Apoptotic Caspases and Genes of Cholesterol Homeostasis in Human Brain
Source: PLoS One. 2014 Oct 16;9(10):e110610. doi: 10.1371/journal.pone.0110610 (PMC4199739; doi:10.1371/journal.pone.0110610)

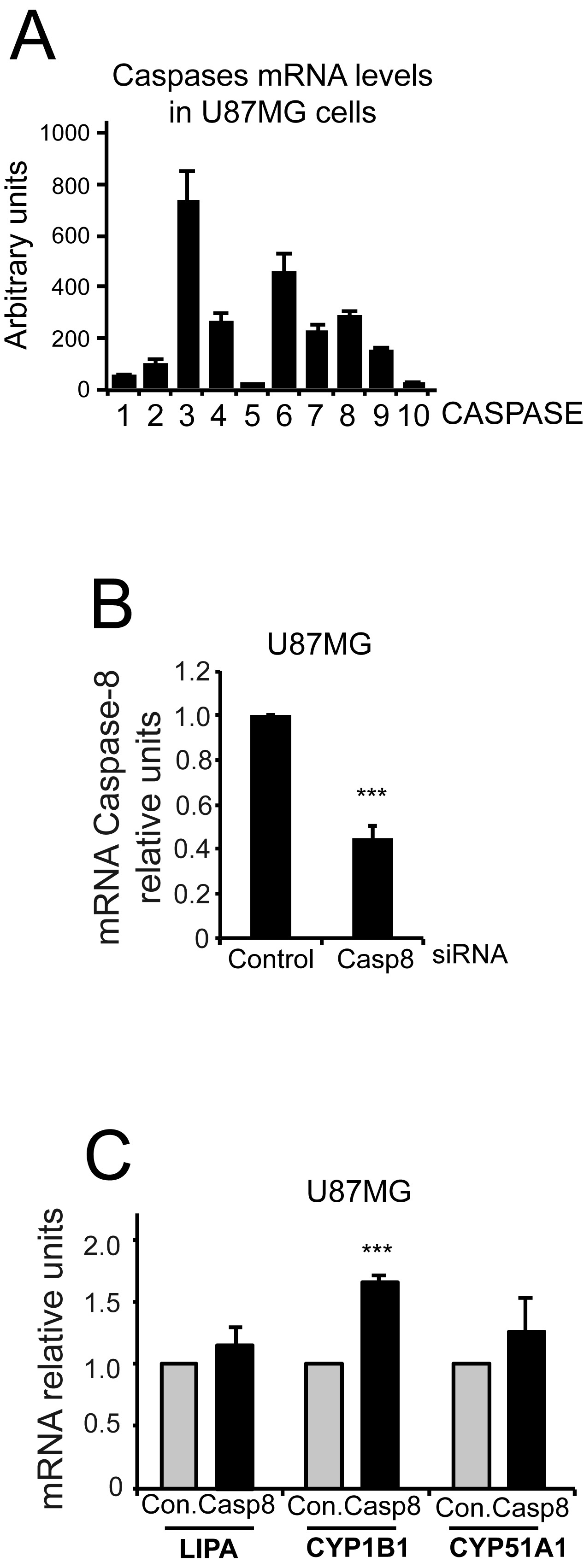

Supplement: Figure S1 — A. Expression profiles in U87MG cells of the different caspases. Microarray expression data were obtained from GSE14889 [118]. B. mRNA expression levels of CASP8 were measured using qRT-PCR in U87MG cells transfected with siRNA against Caspase-8 or a control siRNA. Data are presented as mean ± SD; n = 3. C. mRNA expression levels of CYP1B1, CYP51A1 and LIPA were measured using qRT-PCR in U87MG cells transfected with siRNA against Caspase-8 or a control siRNA. Data are presented as mean ± SD; n = 3. (JPG) [file pone.0110610.s001.jpg]
